# The Promise and Perils of Oncology Care in Medicare Advantage

**DOI:** 10.1001/jamanetworkopen.2024.34650

**Published:** 2024-09-03

**Authors:** Michael Anne Kyle, Nancy L. Keating

**Affiliations:** Department of Health Care Policy, Harvard Medical School, Boston, Massachusetts (Kyle, Keating); Division of General Internal Medicine, Brigham and Women’s Hospital, Boston, Massachusetts (Keating).

The design of the traditional Medicare benefit has changed very little since its inception in the 1960s. Enrollees in traditional Medicare can seek care from any clinicians, practices, or health care organizations (hereafter, providers) enrolled in the Medicare program and have unrestricted access to Medicare’s covered services. While these benefits are appealing, beneficiary cost sharing is often high. Medicare Advantage addresses limitations of traditional Medicare by reducing cost sharing and setting maximum out-of-pocket cost caps, while also offering additional services (eg, vision, dental). Medicare Advantage plans can provide more generous benefits because they apply strong benefit design policies, including constraining their provider networks and imposing coverage restrictions (such as prior authorization).

As enrollment in Medicare Advantage increases—now more than 50% of Medicare beneficiaries are enrolled in Medicare Advantage—researchers have sought to understand the strengths and weaknesses of traditional Medicare and Medicare Advantage and their implications for patients. Elsewhere in *JAMA Network Open*, Kalidindi and colleagues^1^ compared resource use and quality of care for Medicare Advantage vs traditional Medicare enrollees who were undergoing chemotherapy for cancer. They found lower resource use in Medicare Advantage that was primarily associated with savings on physician-administered chemotherapy due to using less expensive chemotherapy regimens and fewer chemotherapy administration visits.

The very high and rising spending on oncology drugs relative to other drugs^2^ may provide an opportunity to improve the value of care, particularly given the substantial variation in prices for different oncology drugs. Research has demonstrated that plan and patient spending for different guideline-concordant regimens of similar efficacy differs by orders of magnitude.^3^ In recent years, spending on infused and oral anticancer drugs accounted for approximately two-thirds of all spending for patients undergoing chemotherapy.^4^ If Medicare Advantage plans can prompt the use of lower-priced drugs, they may realize substantial savings.^5^

Under fee-for-service compensation models, oncologists are reimbursed for physician-administered drugs based on a percentage of the drug’s cost (6% of the average sales price for Medicare). To counteract this incentive to use higher-priced drugs, Medicare Advantage is equipped with utilization management tools. Medicare Advantage plans can—and do—require prescribers to submit prior authorization requests before the plan will approve treatment coverage. They also may implement step therapy and quantity limits. Plans’ use of utilization management is increasing, in part due to limited alternatives to control the high prices of drugs and other services. Carefully considered prior authorization policies may be an important tool in improving cancer care value given the extraordinarily high cost of cancer drugs, the wide variation in costs of guideline-supported treatment, and the urgent problem of financial toxicity for cancer patients.

Utilization management can reduce resource use, but at a cost. Prior authorization processes impose administrative burdens that may limit access to potentially important medications (not assessed in this study, which included patients already receiving chemotherapy), and it leads to delayed or discontinued care among patients already taking cancer drugs.^6,7^ Moreover, clinician burnout is a worsening challenge, and physicians are distressed by the rising administrative burden of prior authorization. This dynamic is concerning for patient care in the longer term. Innovation in the design of Medicare coverage for all beneficiaries remains a policy priority. For example, alternative payment models, such as the Oncology Care Model (OCM), have incorporated incentives to use less expensive drugs. OCM practices saw swifter uptake of biosimilar therapies and higher-value use of supportive care drugs,^4^ suggesting there may be tools to influence prescribing that are more clinician-friendly than prior authorization. The fewer chemotherapy administration visits for Medicare Advantage beneficiaries observed by Kalindi and colleagues^1^ raise questions as to whether plans required less frequent dosing schedules or prioritized chemotherapy regimens with less frequent administration schedules; this is an important question for future study.

Another important strategy used by Medicare Advantage plans to lower resource use is defined provider networks. Medicare Advantage plans may seek to steer patients to providers with less-resource intensive practice styles. Alternatively, such networks could result if providers find the utilization management practices of Medicare Advantage plans too onerous. Evidence from Medicaid demonstrates that greater administrative burdens discourage providers from participating in the program.^8^ Network adequacy for specialty care is already a challenge in Medicare Advantage, especially for rural beneficiaries.^9^ If oncology practices withdraw their participation, patients with cancer will have limited access to care.

Kalidindi and colleagues^1^ found less resource use for Medicare Advantage enrollees undergoing chemotherapy without evidence of negative impacts on quality. Nevertheless, as they acknowledge, the quality measures assessed have limitations. Four of the 5 measures examine some form of utilization. Given that Medicare Advantage already has strong incentives to avoid overuse of care, measures assessing underuse of recommended cancer care might be more relevant, even if few such measures can be studied well with administrative data. In addition, although the authors examined survival—an important outcome measure—this measure was conditional on chemotherapy initiation. If Medicare Advantage limits access to chemotherapy for certain populations (eg, patients with worse functional status or who have more advanced cancers), then a finding of similar survival would reflect worse care in Medicare Advantage.

Despite its growing prominence in the Medicare program, relatively little is known about Medicare Advantage. Whereas traditional Medicare claims data have been foundational to health policy research for decades, Medicare Advantage encounter data have only recently been made available to researchers. Because Medicare Advantage plans are paid via capitation, bills for individual services are not submitted directly to the Centers for Medicare & Medicaid Services (CMS) as they are for traditional Medicare. As such, Medicare Advantage Research Identifiable Files do not yet provide a complete accounting of all spending and utilization by Medicare Advantage beneficiaries. As have other researchers, Kalindi and colleagues^1^ mitigated limitations of encounter data by restricting their sample to the 83 of 380 Medicare Advantage plan contracts with highly complete data submissions. Nevertheless, some of the differences observed could be due to missing data, as the definition of highly complete included plan contract with up to 10% missing inpatient claims.^10^ As the quality and completeness of this data source improves over time with enhanced transmission of data from plans to CMS, findings and our understanding of the association of Medicare Advantage with care delivery may evolve.

This study^1^ provides new evidence about the potential for Medicare Advantage to prompt potentially higher-value care for patients undergoing chemotherapy. Nevertheless, additional work is needed to improve the quality of encounter data, and additional research is needed to ensure that the lower resource use—if not due to incomplete claims—did not come at the expense of less guideline-recommended care for Medicare Advantage beneficiaries. Importantly, this study examines patients who have initiated chemotherapy. Critical questions remain about whether Medicare Advantage vs traditional Medicare beneficiaries experience differences in cancer diagnosis; access to high-quality oncologists, organizations, and clinical trials; and initiation of guideline-recommended treatments.
